# Phloem parenchyma transfer cells in *Arabidopsis* – an experimental system to identify transcriptional regulators of wall ingrowth formation

**DOI:** 10.3389/fpls.2013.00102

**Published:** 2013-04-24

**Authors:** Kiruba S. Arun Chinnappa, Thi Thu S. Nguyen, Jiexi Hou, Yuzhou Wu, David W. McCurdy

**Affiliations:** School of Environmental and Life Sciences, The University of Newcastle, CallaghanNSW, Australia

**Keywords:** *Arabidopsis*, phloem parenchyma, transfer cells, wall ingrowths, transcription factors

## Abstract

In species performing apoplasmic loading, phloem cells adjacent to sieve elements often develop into transfer cells (TCs) with wall ingrowths. The highly invaginated wall ingrowths serve to amplify plasma membrane surface area to achieve increased rates of apoplasmic transport, and may also serve as physical barriers to deter pathogen invasion. Wall ingrowth formation in TCs therefore plays an important role in phloem biology, however, the transcriptional switches regulating the deposition of this unique example of highly localized wall building remain unknown. Phloem parenchyma (PP) TCs in *Arabidopsis* veins provide an experimental system to identify such switches. The extent of ingrowth deposition responds to various abiotic and applied stresses, enabling bioinformatics to identify candidate regulatory genes. Furthermore, simple fluorescence staining of PP TCs in leaves enables phenotypic analysis of relevant mutants. Combining these approaches resulted in the identification of GIGANTEA as a regulatory component in the pathway controlling wall ingrowth development in PP TCs. Further utilization of this approach has identified two NAC (NAM, ATAF1/2 and CUC2)-domain and two MYB-related genes as putative transcriptional switches regulating wall ingrowth deposition in these cells.

## INTRODUCTION

The plant cell wall profoundly defines cell shape and functioning. This observation is particularly acute for transfer cells (TCs) which develop extensive wall ingrowths to aid nutrient transport. These cells *trans-*differentiate from various differentiated cell types at sites where nutrient distribution pathways encounter apoplasmic/symplasmic discontinuities (Pate and Gunning, 1969; Offler et al., 2003). The increase in plasma membrane surface area resulting from wall ingrowth deposition enables increased densities of nutrient transporters to facilitate localized flux of nutrients across these apoplasmic/symplasmic junctions.

Transfer cells are prominent at anatomical sites required for phloem loading and post-phloem unloading processes. In species that perform apoplasmic phloem loading, vascular cells adjacent to sieve elements (SEs) often develop extensive wall ingrowths. Well-known examples include companion cells (CCs) in pea (Gunning and Pate, 1969; Henry and Steer, 1980; Wimmers and Turgeon, 1991), phloem parenchyma (PP) in *Arabidopsis *(Haritatos et al., 2000; Amiard et al., 2007), and both CCs and PP in *Senecio vulgaris *(Pate and Gunning, 1969; Amiard et al., 2007). In pea, the onset of assimilate export from young leaves coincides with the differentiation of leaf minor vein TCs (Gunning and Pate, 1974), and in *Arabidopsis*, sucrose export from leaves is affected if wall ingrowth abnormalities occur in the PP TCs (Maeda et al., 2006). TCs are also commonly observed in cells involved in post-phloem unloading pathways (Patrick, 1997), particularly in seed of cereal crops such as wheat and barley (Thompson et al., 2001). Wall ingrowth formation therefore plays an important role in efficient phloem loading and post-phloem unloading strategies in many species, however, the genetic pathways which regulate wall ingrowth deposition in TCs remain largely unknown.

Transfer cell development occurs across normal developmental windows but also in response to biotic and abiotic stress (Offler et al., 2003). Recent studies using epidermal TCs of *Vicia faba *cotyledons have established that auxin (Dibley et al., 2009), ethylene (Zhou et al., 2010; Andriunas et al., 2011), and reactive oxygen species (ROS; Andriunas et al., 2012) function as inductive signals for TC development. Furthermore, expression profiling of epidermal TCs of *V. faba *cotyledons (Dibley et al., 2009) and endosperm TCs in barley (Thiel et al., 2008, 2012) indicates that wall ingrowth deposition involves differential expression of hundreds of genes. The missing link in this developing molecular understanding of TC biology, however, is the identity of key transcriptional regulators which respond to inductive signals and switch on the downstream cascades of gene expression required to build wall ingrowths. A genetic approach is well-suited to identify such transcription factors. In this mini-review we discuss the features of PP TCs in *Arabidopsis *that enabled a combined bioinformatics and reverse genetics approach to be undertaken to discover that *GIGANTEA *(*GI*)**is a component of a pathway regulating wall ingrowth deposition in PP TCs. Further, we describe preliminary results using this approach to identify previously uncharacterized members of the NAC (NAM, ATAF1/2 and CUC2)-domain and MYB-related gene families as putative transcriptional regulators of wall ingrowth deposition in PP TCs.

## PHLOEM PARENCHYMA TRANSFER CELLS IN *Arabidopsis*

Transfer cells in *Arabidopsis* are known to occur in PP of the minor vein network in both leaves (Haritatos et al., 2000) and sepals (Chen et al., 2012). These PP TCs are defined as Type B TCs (Gunning and Pate, 1969), characterized by having bulky wall ingrowths predominantly abutting SEs and to a lesser extent CCs (Haritatos et al., 2000; Amiard et al., 2007). These three cell types together constitute phloem tissue of the minor vein in *Arabidopsis*, with proportionate numbers of cells of each type relatively consistent throughout the vein system regardless of vein order (Haritatos et al., 2000). SEs are smaller than CCs, as is typical of collection phloem described by van Bel (1996), and PP cells are larger than CCs (Haritatos et al., 2000). Vein order in *Arabidopsis *leaves typically extends to three or four (Haritatos et al., 2000) or sometimes to five orders (Kang et al., 2007). This number is lower than the typically six or seven vein orders seen in most dicot species, and may in part account for the suggestion that both major and minor veins, being in close proximity to mesophyll tissue, are likely to be involved in phloem loading and thus functionally defined as “minor veins” (Haritatos et al., 2000).

A role for PP TCs in phloem loading is based on structural and molecular observations. Prominent symplasmic connections occur between PP and neighboring bundle sheath cells (Haritatos et al., 2000), providing a symplasmic delivery pathway for sucrose from photosynthetic mesophyll cells. Prominent wall ingrowths deposited adjacent to abutting cells of the SE/CC complex infers that the symplasmically delivered sucrose is effluxed across the plasma membrane of PP TCs into the apoplasm (Amiard et al., 2007). Subsequent movement of sucrose into the SE/CC complex occurs via carrier-mediated uptake by SUC2, a sucrose/H^+^ co-transporter localized to the plasma membrane of CCs in *Arabidopsis *(Truernit and Sauer, 1995; Gottwald et al., 2000). The machinery responsible for sucrose efflux from PP TCs into the apoplasm was recently identified as members of the AtSWEET family of sugar transporters (Chen et al., 2012). AtSWEET11 and 12 function as sucrose uniporters that facilitate sucrose efflux, and both localize to the plasma membrane of PP TCs (Chen et al., 2012). An *atsweet11 atsweet12* double mutant showed various physiological traits consistent with impaired sucrose export from leaves (Chen et al., 2012). These authors concluded that PP TCs participate in a two-step phloem loading strategy in *Arabidopsis* – unloading of sucrose from PP TCs into the apoplasm, followed by active uptake of this apoplasmic sugar into the SE/CC complex by SUC2. Interestingly, Chen et al. (2012) propose that the highly localized deposition of wall ingrowths in PP TCs adjacent to cells of the SE/CC complex enables restricted delivery of sucrose into the apoplasm, thus potentially reducing access to this apoplasmic sugar by pathogens. Others have suggested that the extensive deposition of bulky and highly localized wall ingrowths in PP TCs adjacent to SEs provides a physical barrier to protect against infection by pathogens which commonly target PP cells as an entry point into the vascular network (Amiard et al., 2007).

Haritatos et al. (2000) observed that PP TCs also form asymmetric plasmodesmatal connections with adjacent CCs in *Arabidopsis *veins, implying that phloem loading in this system may also occur passively via plasmodesmatal pathways under certain physiological conditions. This observation implies that phloem loading strategies in different scenarios may be developmentally plastic, switching alternately from active, apoplasmic loading, to passive, symplasmic loading, even along a single vascular bundle (Slewinski and Braun, 2010). The molecular signals that may control such plasticity are unknown, however, the identification by Chen et al. (2012) that the promoter for *AtSWEET11* drives expression in leaf tissue specifically in PP cells provides a valuable addition to the molecular tool box to investigate such processes.

## *Arabidopsis* PHLOEM PARENCHYMA TRANSFER CELLS AS AN EXPERIMENTAL SYSTEM TO INVESTIGATE GENETIC CONTROL OF WALL INGROWTH DEPOSITION

Importantly for genetic analysis of TCs in a model species, wall ingrowth deposition in *Arabidopsis *PP TCs is responsive to various stresses. The extent of wall ingrowth invaginations in PP TCs of leaf minor veins was significantly increased in response to stress caused by high-light or exposure to methyl jasmonate (Amiard et al., 2007). Furthermore, the high-light response was reduced in the jasmonate-deficient double mutant *fad7-1 fad8-1* (Amiard et al., 2007), implying the unexpected conclusion that chloroplast-derived jasmonates signal wall ingrowth deposition in PP TCs in response to oxidative stress. In support of this conclusion, a *npq1-2 lut2-1 *double mutant showed increased levels of wall ingrowth deposition compared to wild-type when subjected to high-light stress (Demmig-Adams et al., 2013). The double mutant lacks zeaxanthin and its isomer lutein, photoprotective agents which suppress lipid peroxidation and most likely oxylipin (methyl jasmonate and its precursors jasmonic acid and 12-oxo-phytodienoic acid) formation. The absence of this suppression in the *npq1-2 lut2-1* double**mutant presumably leads to higher levels of jasmonic acid when plants are switched from low to high light, thus the observed increase in deposition of wall ingrowths in PP TCs (Demmig-Adams et al., 2013).

Wall ingrowth deposition in PP TCs is also responsive to cold stress. As part of their study investigating the role of tocopherols in photoprotection, Maeda et al. (2006) reported that growth of wild-type plants at low temperature caused increased deposition of polarized wall ingrowths in PP TCs. In contrast, at low temperature the vitamin E-deficient mutant, *vte2*, displayed greatly increased levels of abnormal wall ingrowth deposition, including loss of polarized deposition and substantial accumulation of callose in and around the wall ingrowths (Maeda et al., 2006). Not surprisingly, the *vte2* plants showed reduced sugar export and consequently increased levels of soluble sugar in leaves of cold-treated plants (Maeda et al., 2006). This result indicates not only that low temperature in itself causes increased wall ingrowth deposition, but at low temperature the signal(s) causing localized wall ingrowth deposition are lost or over-ridden in the *vte2 *mutant. Irrespective of this issue, however, the study by Maeda et al. (2006) adds low temperature to high-light and exposure to methyl jasmonate (Amiard et al., 2007) as stress signals causing wall ingrowth deposition in *Arabidopsis *PP TCs. From the perspective of identifying transcriptional regulators of wall ingrowth deposition, the importance of these observations is that they enable bioinformatics approaches to be used to identify candidate genes.

## FLUORESCENCE STAINING OF PHLOEM PARENCHYMA TRANSFER CELLS IN *Arabidopsis* LEAVES

**FIGURE 1:**
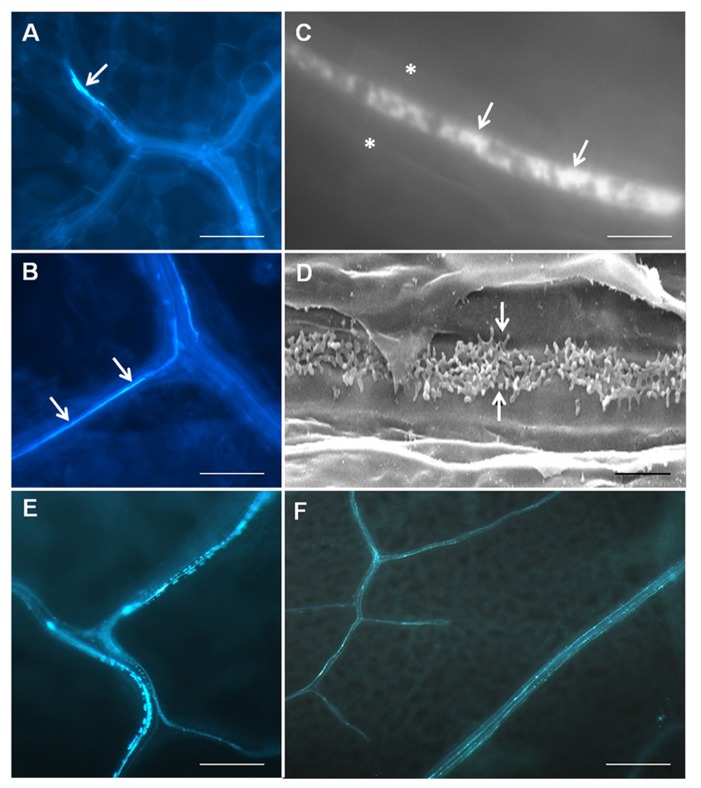
**Imaging of PPTCs in *Arabidopsis* veins using fluorescence staining and scanning electron microscopy.** Calcofluor White staining of cleared leaf tissue (**A**–**C**) showing presence of PP TCs in a terminating minor vein (arrow in **A**) and as more continuous linear strands of staining running along major veins (arrows in **B**). Higher magnification reveals a central band of mottled fluorescence (arrows in **C**, asterisks mark cell edges) in a PP TC which corresponds to the deposition pattern of reticulate wall ingrowths seen by scanning electron microscopy in these cells (arrows in **D**). Staining of PP TCs by aniline blue (**E, F**) shows the same patterns of staining as revealed by Calcofluor White, albeit with superior signal-to-noise properties (see **F**). Punctate staining indicating the non-continuous development of PP cells into PP TCs along a given length of vein is particularly evident in **E**. The images in **A**–**D** are reproduced from Edwards et al. (2010) and **E** and **F** are unpublished data. Staining with aniline blue was performed identically to that of Calcofluor White, except that 0.01 (w/v) aniline blue in 70 mM phosphate buffer, pH 8.5, was used to replace 0.05% (w/v) Calcofluor White. Scale bars: **A**, **B**, **E** = 100 μm; **F** = 200 μm; **C** = 5 μm; **D** = 2 μm.

Transfer cells typically occur deep within tissue systems and consequently have mostly been studied by electron microscopy, a process which is not compatible for high throughput genetic screening using *Arabidopsis*. Wall ingrowths lack lignin but are abundant in cellulose and hemicelluloses (DeWitt et al., 1999; Dahiya and Brewin, 2000; Vaughn et al., 2007), therefore Edwards et al. (2010) used Calcofluor White staining of cleared leaf tissue as a means to rapidly assess the abundance of PP TC development across whole leaves. Staining showed strong patches of fluorescence in terminating minor veins but also more continuous, linear regions of fluorescence often seen as one or two rows of staining within each vein (**Figures 1A, B**). Higher magnification views revealed that the Calcofluor White staining showed a distinctive mottled appearance, a characteristic consistent with staining the patchy and tangled wall ingrowths seen in leaf PP TCs by scanning electron microscopy (**Figures 1C, D**; Edwards et al., 2010). The non-continuous staining pattern for PP TCs along a given vein is consistent with observations by transmission electron microscopy that not all PP cells contain wall ingrowths (Amiard et al., 2007), a situation possibly reflecting potential plasticity in phloem loading mechanisms as discussed by Slewinski and Braun (2010). Furthermore, the ability to survey whole leaves for the presence of PP TCs clearly established that these cells are prominent in both minor and major veins of the vascular network, an observation consistent with the conclusion that both vein types in *Arabidopsis *are likely to be involved in phloem loading (Haritatos et al., 2000).

A recent improvement for fluorescence staining to detect PP TCs in *Arabidopsis* leaves has been the use of Aniline Blue rather than Calcofluor White. Callose is an abundant component of the electron translucent outer layer of wall ingrowths in both epidermal TCs of *V. faba *cotyledons (Vaughn et al., 2007) and *Arabidopsis* PP TCs (Maeda et al., 2006, 2008). Other than being deposited in sieve plates, callose is mostly absent from other tissues in unwounded leaves, thus giving superior signal-to-noise staining of PP TCs compared to Calcofluor White (**Figures 1E, F**). Double labeling experiments have shown that Aniline Blue gives the same mottled patterns of staining for PP TCs as does Calcofluor White (J. Hou, unpublished observation and see Edwards et al., 2010), thus confirming that Aniline Blue can be used as a convenient and high throughput fluorescence stain for wall ingrowth deposition in PP TCs.

## IDENTIFICATION OF GIGANTEA AS A COMPONENT IN THE REGULATORY PATHWAY CONTROLLING WALL INGROWTH DEPOSITION IN PP TCs

Combining the experimental features of PP TCs as described above, Edwards et al. (2010) performed a hierarchical bioinformatics analysis of publically available microarray datasets and identified *GI*, a well-known regulator of flowering time (Koornneef et al., 1991; Fowler et al., 1999), as one of about 46 genes commonly up-regulated in leaves subjected to either high-light or cold stress. Phenotypic analysis using Calcofluor White staining of leaves revealed that in both *gi-2 *and *gi-3* plants, the abundance of PP TCs in veins was reduced up to 15-fold compared to wild-type. Over-expression of *GI *in the *gi-2 *mutant background restored PP TC abundance back to wild-type levels, whereas rescue of wall ingrowth deposition in *gi-2 *did not occur after exposure to high-light, methyl jasmonate or cold. Based on these outcomes, Edwards et al. (2010) proposed that *GI* may be regulating wall ingrowth deposition downstream of inputs from stress signals, possibly through detoxification of ROS (see Cao et al., 2006). In epidermal TCs of *V. faba *cotyledons, extracellular H_2_O_2_ is known to act as a polarizing signal to direct aspects of wall ingrowth deposition (Andriunas et al., 2012; Xia et al., 2012). In *Arabidopsis*, however, H_2_O_2_ is abundant in leaf vasculature, even in the absence of stress (Mullineaux et al., 2006), hence its ability to act as a local signal directing polarized wall ingrowth formation in PP TCs needs further investigation.

## IDENTIFICATION OF NAC-DOMAIN AND MYB-RELATED TRANSCRIPTION FACTORS AS PUTATIVE REGULATORS OF WALL INGROWTH DEPOSITION

Based on the successful approach used by Edwards et al. (2010), we recently performed an extended bioinformatics analysis to identify transcription factors commonly up-regulated in leaf tissue in response to high-light, methyl jasmonate, and cold. Phenotypic analysis using Aniline Blue staining of leaves from homozygous T-DNA insertional mutants from this list identified several previously uncharacterized NAC-domain (At3g04420 and At1g33060) and MYB-related genes (At1g25550 and At1g49560) which showed significantly reduced abundance of PP TCs in veins of mature leaves compared to wild-type (**Table 1**). The levels of reduced abundance in each line, while significant, were not comparable to that seen for the *gi-2 *mutant (**Table 1**), indicating the possibility that these transcription factors may be acting redundantly with unidentified orthologs in controlling wall ingrowth deposition. *In silico *expression data (eFP and Genevestigator) shows that all four genes are expressed at very low levels in leaves, and qPCR confirmed this observation directly for both expanding and fully expanded leaves (J. Hou and Y. Wu, unpublished observations). Low expression might be expected for genes operating as putative regulators of wall ingrowth deposition specifically in PP TCs, since the number of PP TCs relative to most other cell types in the leaf is exceedingly low (Haritatos et al., 2000; Edwards et al., 2010), and many plant transcription factors are expressed at low levels (Czechowski et al., 2004). Given these observations, we are using both constitutive (CaMV-35S promoter) and PP-specific (*AtSWEET11 *promoter) over-expression to test the role of these transcription factors as regulators of wall ingrowth deposition in *Arabidopsis*.

**Table 1:** Phenotypic analysis showing reduced abundance of PPTC staining for two NAC-domain and two MYB-related genes identified by bioinformatics as candidate transcriptional regulators of wall ingrowth deposition in PPTCs of *Arabidopsis* leaf veins.

Gene number	Mutant allele	% vein length showing staining for PPTCs^§^
WT (Col-0)		45.3 ± 3.6
NAC-domain		
At3g04420	FLAG_009F02	18.2 ± 3.8**
At1g33060	SALK_085596	27.5 ± 2.9*
At1g33060	SALK_024241	31.5 ± 4.7*
MYB-related		
At1g25550	SALK_144656	16.3 ± 2.4**
At1g49560	SALK_085182	20.5 ± 2.9*
At1g49560	SALK_095775	15.6 ± 2.7**
*GIGANTEA*		
At1g22770	*gi-2*	3.3 ±1.0**^†^

§ This value was measured from mature, Aniline Blue-stained leaves according to our previously published method (Edwards et al., 2010). Data is presented as mean ± SE from two leaves from each of three plants per line.

* *P* < 0.01,

** *P* < 0.001.

† Data for *gi-2 *taken from Edwards et al. (2010).

Interestingly, ectopic over-expression of vascular-related NAC-domain6 (VND6) or VND7, both NAC-domain transcription factors, causes *trans*-differentiation of non-vascular cells into metaxylem- and protoxylem-like vessel elements, respectively (Kubo et al., 2005), a process involving localized secondary wall deposition. Over-expression of various MYB transcription factors such as AtMYB46 (Zhong et al., 2007) and AtMYB83 (McCarthy et al., 2009) also causes ectopic secondary wall formation, leading to the conclusion that hierarchical transcriptional pathways, with NAC-domain and MYB transcription factors acting as either first- or second-tier “master switches,” co-ordinate the gene expression programs required for localized secondary wall deposition (Zhong et al., 2010). Building wall ingrowths in TCs is also an example of highly localized wall deposition (McCurdy et al., 2008), thus our finding that two NAC-domain and two MYB-related genes are putative regulators of this process in PP TCs may indicate evolutionarily conserved roles for members of these two large gene families in regulating transcriptional cascades involved in localized wall deposition. Further support for this proposition comes from the observation that ZmMRP-1, a transcription factor which regulates basal endosperm TC development in maize, is a member of the MYB-related family of transcription factors in plants (Gómez et al., 2002, 2009).

## CONCLUSIONS AND FUTURE DIRECTIONS

The formation of wall ingrowths in TCs impacts on phloem loading and post-phloem unloading processes in many species, with corresponding impacts on plant development and reproduction. Development of an experimental system to investigate PP TCs in *Arabidopsis* has proven useful to identify candidate genes operating as putative transcriptional regulators of wall ingrowth deposition in TCs. The discovery of *GI *as a component in the pathway regulating wall ingrowth deposition, and identification of NAC-domain and MYB-related genes as putative “master switches” involved in controlling this process, provides new lines of investigation to understand the genetic control of TC development and the cell biology of localized wall ingrowth deposition. Ultimately, identifying master switches which respond to various inductive signals to coordinate wall ingrowth deposition in TCs may provide new opportunities for improving crop yield by manipulating this process.

## Conflict of Interest Statement

The authors declare that the research was conducted in the absence of any commercial or financial relationships that could be construed as a potential conflict of interest.
